# Ethical Dilemmas for Dental Students in Greece

**DOI:** 10.3390/dj11050118

**Published:** 2023-05-02

**Authors:** Maria Antoniadou, Evangelia Masoura, Marina Devetziadou, Christos Rahiotis

**Affiliations:** Dental School, National and Kapodistrian University of Athens, Thivon 2, 11527 Athens, Greece

**Keywords:** ethics, dilemmas, dentistry, dental students, undergraduate students, dental education, tooth extractions, bioethics, Generation Z

## Abstract

Professional dental ethics for students are based on promoting oral health for dental patients and reinforcing an anthropocentric approach to communication and dental services. A total of 133 dental students (males 33.8% N1 = 46, females 66.2% N2 = 87) completed the study questionnaire. Descriptive statistics were applied, and non-parametric Kruskal–Wallis tests were used (*p* < 0.05). Students refuse services to patients that misbehave (37.6%), have irrational demands (18%), and when clinical cases exceed their capabilities (36.8%). Of the participants, 50.4% want to waive confidentiality when abuse is declared. Ethical role models are educators (33.8%), other qualified dentists (25.6%), and their parents (21.8%). Female gender positively affects integrity (*p* = 0.046), altruism (*p* = 0.032), and difficulty in conversations among colleagues (*p* = 0.036). Students outside the capital are less interested in esthetic issues (*p* = 0.007), in giving more than one treatment plan (*p* = 0.006), and in being confronted with inadequate treatments from other colleagues (*p* = 0.005). Family income positively affects clinical skills (*p* = 0.003), trust issues (*p* = 0.008), and moral insight and intuition (*p* = 0.02). Presentation with clinical scenarios is the preferred educational method (49.6%). Dental students show compassion for poor patients, respect patients’ autonomy, and guide patients to choose the best treatment plan before receiving dental ethics seminars. There is a positive relationship between the ethical footprints of students and gender, origin, family income, postgraduate studies, and future professional plans. Factors and ways to incorporate ethics in the dental curriculum could be considered when planning relevant courses.

## 1. Introduction

Every day of their professional lives, dentists face decisions that have ethical content and are critical for their sense of duty and professionalism [1]. Professional ethics are based on promoting good for the dental patient and reinforcing an anthropocentric approach to dental communication [2]. It has always been the case that a good dentist should exhibit clinical skills and knowledge and the ability to provide quality care with integrity, communicate effectively and treat patients quickly, fairly, and ethically [3]. It is reported that a healthcare provider can be a true professional with a solid understanding and acceptance of dental ethics [4,5]. In contemplation of dealing with human rights and liberties, every profession, including dentistry, eventually develops a professional code of ethics to achieve responsible behavior from all its members [6,7]. In consequence, all members of the profession should be cognizant of the special rules of customer care and cooperation relating to their profession [8,9]. 

Technological advances and shifts in societal attitudes have created more clinical situations in dentistry that require ethical analysis nowadays [10]. Concerns about the poor quality of care, violations of public trust, flagrant advertising, self-regulation practices, patient autonomy, informed consent, conflicts with patients, interprofessional relationships, and financial interactions are only a part of the relevant issues [11]. How dentists respond to these problems determines the practice’s character, sustainability, and dental education quality [12]. Of course, not all evaluations in everyday dental practice must be judged as moral or ethical. They might also be a matter of personal taste, preference, or beliefs [1,2,3]. However, most people consider an action moral or ethical when it meets one or all of five essential characteristics: ultimacy, universality, altruism, publicity, and ordering [13]. The problem arises when the difference between excellent and evil must be clarified or we must choose between two goods or evils [12,13]. 

In general, “ethics”, as it comes from the Greek word “ethos” or “moral”, derived from the Latin word “mores”, regulates someone’s character and behavior [14]. Humans naturally imply moral values and effective communication rules [15]. In dentistry, a successful dental treatment plan is based on the relationship between the therapist and the patient, which must be governed by mutual trust [2,5,14,16]. Specific values are central to achieving trust in dentistry, such as: 1. Patient’s general health; 2. Patient’s oral health; 3. Patient’s autonomy; 4. The dentist’s preferred patterns of practice; 5. Aesthetic values, and 6. Efficiency in the use of resources [15,17]. The American Dental Association (ADA) [6] has given five fundamental ethical principles: patient autonomy, non-maleficence, beneficence, justice, and veracity, which are explained as follows: (1) Respect for the patient’s autonomy means that the expert should regard self-rules to guide patients into deciding on the right choice that reflects their values and goals [10]; (2) Non-maleficence obliges the practitioner to do no harm and to act according to the patients’ most significant advantage and personality respect [14]; (3) Beneficence encourages dentists to prevent damage, promote health an provide information to balance benefits against risks and costs [16]; (4) Justice supports the fair distribution of the benefits and risks of dental care associated with community interventions that address dignity and sustainability [17,18]; (5) Veracity, finally, highlights the trained consent cycle [19,20,21].

Furthermore, numerous ethical theories exist, but most research highlights five fundamental ones for dental practitioners [10,11,17,18,19]. These theories are the following: (1) Teleology. Acting ethically does not come from a motive or an intention but from the criteria of right and wrong resulting from the act. If the action causes a satisfactory result, it is considered ethical [22]. (2) Deontology. The most acclaimed principle of ethics is the “Golden Rule” in the Bible: “Treat other people with the concern and kindness you would like them to show toward you”. This theory underlines the ethical duty and responsibility that a practitioner must have when making a moral decision, regardless of its consequences [23]. (3) Motivation. Motives are not driven by absolute values but by intentions (motives). The intention behind a specific action should be the basis for understanding whether an act is ethical [10,17]. (4) Right to poise. It supports that patients, patients’ families, and dentists maintain a choice of respect during dental care collaboration [18,19]. (5) Natural law theory. Ethical standards are derived from the nature of the human being and the nature of the world. It highlights the result [24]. (6) Transcultural ethical theory. The need to include cultural factors in resolving an ethical dilemma is essential due to the diversity of ethical values and morals [25]. The advantage of the cross-cultural ethical system is that it crosses other ethical theories and joins consideration of differences in dental decision-making and individual cultures [19,20,21]. In the relevant literature, though, it is mentioned that ethical theories do not answer the ethical dilemmas that dentists might encounter [19,20,21,26]. Instead, they provide a framework for making ethical decisions by the practitioner when combined with critical information received by the patient [21,26]. That is where the dental code of ethics should fill the gaps. 

The dental educational program, in addition to offering the necessary clinical and basic science knowledge, technical competencies, and clinical skills, should also inculcate the values and attitudes of the profession. It would also be helpful for students to know how moral claims are justified and what some prominent positions are in dental ethics nowadays, nationally and worldwide. To begin with, the Hippocratic Oath and the National Dental Code of Ethics [6] can be a good support for making ground decisions in dentistry. Bioethics, referring to the application of ethical principles in health care, is a branch of applied ethical theory, and it is as old as the Hippocratic Oath, “Primum non-nocere”, which suggests “no harm” [1,6,7]. Legislatures enact laws when there are repeated failures to adhere to ethical standards. Although these must be known to dentists by dental schools, and even though 80% of all dental schools nowadays offer courses in professional ethics [27], many emphasize jurisprudence [13]. Additionally, the ability of these courses to stimulate valid ethical reasoning may be of concern because few of the faculty have formal training in ethics and relevant law [14]. 

Further, progress in dental research has brought critical ethical issues in modern dentistry. Therefore, nowadays, dental professionals and students face different moral conflicts in their clinical practice than before [14,28]. Hence, there is an imperative need for the dental profession to acknowledge the role that ethics play in the legitimacy of the profession. Most professional societies or national organizations have established ethical guidelines for their members concerning human dignity, confidentiality, privacy, and patient rights [1,5,21]. However, the dental curriculum has an overloaded schedule for students, leaving no time for the trainees to make decisions for their clinic patients [29,30]. Furthermore, there is no time to evaluate the student’s level of ethical development, nor is there, in most curriculums, a particular subject designed to help students in ethical decision-making processes and approaches. In the best scenario, one or two seminars are assigned to dental ethics during the dental curriculum, which is also the case in the Dental School of the National and Kapodistrian University of Athens, Greece. However, there are several seminars in clinical decision-making for therapeutic plans throughout the undergraduate program. This is in contrast with the fact that the students are considered to exhibit not only professional behavior but also moral responsibility in a minimal amount of time and mainly without the necessary educational background [9,11,18].

As known, current 5-year undergraduate dental students are Generation Z’s future dental practitioners, meaning people that have mid-to-late 1990s birth years [31]. Generation Z (or more commonly, Gen Z for short), are children of Generation X (people born between 1965 and 1981) and have specific characteristics that determine their behavior [32]. Thus, the current study aims to understand the ethical dilemmas these Gen Z students face during their clinical training concerning patient communication issues, colleagues’ relationships, ethical values, and models supporting their decisions and future educational and professional plans. Further, the scope is to examine whether specific demographic characteristics influence their ethical behavior and clarify the ethical trends they address. The null hypothesis of the present study is that there are no differences between sexes, place of origin, family income, and future educational and professional plans among 5th year undergraduate dental students in Greece.

## 2. Materials and Methods

For this study, a digitally formed questionnaire designed in google forms was distributed to the 5-year undergraduate dental students attending the clinical program at the National and Kapodistrian University of Athens, Greece, dental school. The completion took place on 20 November 2022, during a 2-h seminar of the course “Professionalism and Management of dental office”, a compulsory subject of the dental curriculum of the school. The students had 15 min to complete the form. They had neither heard anything about the issues addressed in the questionnaire nor anything about the Code of Ethics issued nationally. All present students were given the same access and the same opportunity to complete the questionnaire. No reward was given for participating in the study.

A panel of 3 professors confirmed the validity of this questionnaire, all experts on dental education, and members of the school’s curriculum committee who reviewed and revised the survey questions to be relevant to the topic and expressed correctly. They worked independently and then in two joint meetings with the authors to make final suggestions. The questionnaire was further validated by fulfillment of it by ten members of the academic staff and ten postgraduate students that were not involved in the study. Finally, the accuracy of the completion was checked by making all questions obligatory to submit the questionnaire, while submission was only allowed once. 

The online questionnaire included a short introductory message describing the purpose of the study and stressing voluntary participation, confidentiality, and the right to refuse participation. Consent was obtained by asking participants to confirm that they agreed to complete the questionnaire by marking a “Yes, I agree to participate” box. Ethical approval was obtained from the Ethics Board of the dental school, and the study proposal was registered under the number 395/20 April 2022. A QR code was assigned to the questionnaire link to provide direct access through students‘ smartphones. 

The questionnaire consisted of (a) demographic characteristics (personal information regarding age, gender, family income, and place of origin); (b) five ethical dilemmas mentioned as statistically significant in previous studies [9,11,18]; (c) ten close-ended clinical scenarios where students could select the most suitable answer from the ones provided. The answers provided were meant to challenge the responder’s ethical thinking; (d) seventeen ethical challenges to be evaluated by frequency (never, rarely, often) and fifteen values to be considered according to importance (the least important up to the most important); and (e) an open-ended question addressing suggestions on the methodology of teaching of dental ethics. The survey guaranteed anonymity to respondents (Appendix A).

Descriptive statistics were applied, and non-parametric Kruskal–Wallis tests were used to examine group differences. All reported probability values (*p* values) were compared with a significance level of 5% (*p* < 0.05). The analysis of coded data was performed using IBM SPSS Statistics for Windows, Version 26.0 (SPSS Inc., Chicago, IL, USA).

## 3. Results

In this cross-sectional study, 5-year undergraduate dental students in Athens, Greece, filled out the questionnaire (133 of 138 students, response rate 95.6%). The average age was 24 ± 2 years, and 33,8% were males (Ν1 = 46) while 66.2% were females (Ν2 = 87). Out of the 133 students, 48.1% originated from Athens. In contrast, the others were split between other urban cities in Greece (12.8%), islands (9.8%), and the countryside (21.1%), while 8.3% of the participants reported other countries as their origin. Of the students, 21.8% did not mention their family income (either not knowing or replying that they did not care), while 14.3% reported income below 15,000 EUR/year, 35.3% mentioned family income below 35,000 EUR/year, 15.8% between 35,001–50,000 EUR/year and 12.8% reported above 50,000 EUR/year. It was also observed that females (32.9%) did not seem aware of their family income. Further, 40.6% claimed they would attend an MSc program after their degree, and 6% of participants mentioned that they would not work in dentistry (Table 1).

When students were asked about the most challenging ethical dilemma, 23.3% suggested that the most important one is to offer dental services to patients with contagious diseases, followed by 19.6% who declared fee-related reasons according to which they prefer extractions instead of endodontic treatment and 18.8% who reported patients’ neglect by caregiver or family. Finally, 11.3% expressed their quandary about revealing terrible news regarding negative clinical findings to a patient. Further, students were asked when a dentist should refuse their services. The results were split between patients that show no respect and misbehave (37.6%), cases where dental operations exceed the capabilities of the therapist (36.8%), and situations where patients insist on irrational treatments/solutions (18%). When asked the students’ opinion on what circumstances the patient–therapist confidentiality can be waived, 50.4% of the participants declared an abuse incident.

In comparison, 27.8% claimed this could not occur only under prosecutorial intervention. Additionally, students see as ethical role models their educators (university associates/professors) (33.8%), other qualified dentists (25.6%), and finally, their parents (21.8%). When asked about educational tools and methods for being educated on ethical issues, students declared that presentations with cases involving ethical issues would be the best way (49.6%). In comparison, 25.6% would like to work on close-ended (multiple choice) questions based on the national code of dental ethics and 15% would prefer group role-playing. 

The second part of the questionnaire offered a list of clinical scenarios, prompting students to evaluate whether the result was based on ethical standards, judge the outcome, or offer a solution. The students showed compassion for patients who could not immediately respond to treatment fees, respect for their autonomy, and guided the patient to choose the best treatment for them. More specifically, 89.5% suggested that the dentist has the right to refuse to provide services to a patient that insists on mass extraction of their teeth against the conservative treatment that the dentist recommends to them. In the case of a patient wanting all their remaining teeth extracted to put on complete dentures, 51.9% believe that raising patients’ awareness of dental issues will provide better results for the patient’s health. Further, 78.2% suggested that in the case of a 15-year-old pregnant patient, they would not talk to her parents, urge her to talk to them herself and delay the treatment as much as they can.

Further, in the case of a 65-year-old active and good-looking man who wants “the whitest the better” for the teeth of his new dentures, 59.4% of the students suggested mutual collaboration for a commonly accepted color choice. If the dentist gives the patient only one treatment option, 61.7% of the students suggested that the dentist explain the different options in his treatment plans and guide him to the right choice. If patients have economic issues with fulfilling their emergency therapy, 42.1% would only complete the endodontic treatment and facilitate the payment for the patient. In comparison, 33.1% of them would complete the entire treatment plan facilitating the patient to pay when they can. Then, in the case of a disappointed and angry patient who, after three years, wants to have the prosthetic work replaced pro bono, 40.6% of the students suggested that the patient must generally pay for the construction of a new bridge, while for those refusing to put the prosthetic work that the dentist has made because they cannot speak and do not like them aesthetically, 54.9% would make an effort to modify them to meet the objections of the patient. Additionally, 17.3% of the students would tell the patient that the dentist has been charged for the work and that the patient should pay even if he does not want them. 

In Table 2, common ethical dilemmas addressed by students are presented. The data reveal that 49.6% of the students regularly face substantial treatment from another colleague and 35.3% of the students often need to correct them. However, the students rarely disagree with treatments provided by other colleagues (57%) and prefer to avoid explaining treatment errors to their patients (49.4%). “Never” was chosen as an answer from most of the students when they were questioned about having to criticize another colleague’s work (72.2%), over-treating a patient (60.9%), dealing with a patient who is insisting on inappropriate treatments, and failing to address ethical challenges (58.68%). In addition, the violation of medical confidentiality was never addressed as an ethical challenge by 73.3% of the responders. The questionnaire also revealed that the students rarely face practices/policies inconsistent with the standards of care (44.4%), dealing with patients unable to come up with a decision on their own (48.1%) or patients who refuse to accept the treatment plan (43.6%). Rarely, students must also notify a patient about a malignancy (48.1%), encounter a patient who blames another colleague for their prices (38.3%), and finally run into a situation where the patient cannot pay for their treatment (48.1%). 

The responders in the third part of the questionnaire were asked to evaluate from a list provided the most important values (Table 3). The results showed that the essential values in hierarchical order were mutual trust (83.5%), individual clinical practice (81.8%), integrity (74.7%), justice (74.4%), compassion (66.2%), and altruism (65.4%). Conversely, the least significant values according to the responders were voluntary actions (9%) and the service of personal needs (13.5%). 

Table 4 presents the multivariate analysis of responses collected by the respondents compared to different demographic factors such as gender, origin, family income, future MSc studies, and future professional plans. Issues related to gender seemed statistically significant in most of the critical values mentioned by the responders. Integrity (*p* = 0.046) and altruism (*p* = 0.032) showed the most significant difference between the sexes. Difficulty in conversations amongst colleagues appeared to also be affected by gender, with females correlated positively (*p* = 0.036). Ethical dilemmas regarding the dentist–patient relationship (*p* = 0.07), the service of personal needs (*p* = 0.04), and moral courage (*p* = 0.039) were affected by the origin of the responders. The interaction with other medical professions (*p* = 0.03) and ethical insight and intuition (*p* = 0.02) are the most affected by the family income of the responders. Furthermore, students who planned to attend MSc studies expressed more profound concern for patients who refuse to follow the treatment (*p* = 0.047) and the trust between patients and health care providers (*p* = 0.039). 

## 4. Discussion

The 5-year undergraduate dental students in Athens, Greece, are all members of Gen Z, meaning they were born between 1995 and 2010 [31,32]. As members of this generation, they were exposed to the internet from their earliest youth, resulting in a hypercognitive generation very comfortable with collecting and cross-referencing many sensible sources of information by integrating virtual and offline experiences that create a specific profile different than other generations [33,34]. Gen Z behaviors are based on individual expression and motivation for various ethical causes towards which they make decisions and relate to others in a highly analytical, accurate, and pragmatic way [35]. Moreover, they profoundly believe in the efficacy of dialogue to solve conflicts [36], improve the world [31], and share the truth and freedom of expression [32] by opening themselves to understanding different kinds of people [36].

In our study, 5-year undergraduate dental students of Gen Z in Athens, Greece, reacted with bigger or smaller similarities to the findings of relevant literature regarding the ethical behaviors of people of their generation. To start with, the most challenging ethical dilemmas for them are offering dental services to patients with contagious diseases (23.3%) (this can be attributed to the ongoing COVID-19 pandemic), poor patients (19.6%) (as they sympathize with patients unfavored situation), and patients with poor oral condition due to caregiver neglect (18.8%) (as they are more sensitive to racism, violence, neglect, and bullying behaviors) [32,37]. They also favor refusing dental services to patients that misbehave (37.6%) and have irrational demands (18%) for the same reasons. As they stand for truth and transparency and value authenticity [31], it is not surprising that in our study, they expressed the will to avoid getting involved in clinical cases that exceed their capabilities (36.8%). Elsewhere, it has been reported that out of thousands of ethical issues surrounding the dental profession and the patient–dentist relationship specifically, the most important ones are the patient’s autonomy, poor treatment by another colleague, refusal of treatment, confidentiality issues, and the dentist–patient relationship [10]. Our study never addressed violating medical confidentiality as an ethical challenge (73.3%).

In comparison, 60.9% of the students had never had overtreatment issues to address, in contrast with findings elsewhere that agree that overtreatment is an ethical issue and dentistry, in general, is susceptible to that, although it is considered highly unethical [29]. The fee-related dilemmas of our students were also reported elsewhere with more severe acknowledgment. In the study of Maybodi et al. [28], the authors reported that the most common challenges faced by dental students were compromising treatment due to cost issues (77.1%), a percentage almost triple the one reported here. This can be attributed to the chronological challenges of the current worldwide pandemic, cultural differences, and Gen Z characteristics [31,32,36]. The same results were reported where undergraduate, postgraduate, and house surgeons were compared. Only 25% of undergraduates and 21% of participants expressed fee-related dilemmas; the most significant issue mentioned by 75% of the undergraduate students were treatment-related problems [37]. As reported in a survey by Reis et al. [35], students identified poverty as a distant issue and addressed the responsibility of the government or the poor individuals themselves. Additionally, the students preferred not to work with patients living in poverty in the future, and they claimed that the only way to treat those patients was through volunteering [38]. Our data revealed that our students showed compassion for patients with economic issues and were willing to help by doing part of the treatment until the patients could find ways to pay for the services. 

In this study, we also addressed the issue of whether a dentist has the right to refuse treatment. Of all participants, 98.4% said that the dentist has the right to refuse to provide care, while elsewhere, 50% of the participants claimed that the dentist should refuse treatment [34]. In comparison, only 18% of the postgraduate students claimed the same, and 82% of the dentists, while only 16% of the participants believed this decision would be wrong, stubborn, or show indifference [39]. In accordance, 66% of the students and 89.8% of the dentists in the study of Maybodi et al. [28] expressed the right of the clinician to refuse treatment.

Considering the multivariate effect of different factors affecting 5-year undergraduate dental students’ response to ethical dilemmas, we should mention that ethical differences rarely are correlated with gender in studies on health professionals [10]. However, in a study by Seo et al. [40], a statistically significant difference was observed in factors related to gender, age, religion, spouse status, and clinical career. In our study, the female gender was positively related to values such as integrity and altruism while expressing difficulty in conversations among colleagues. The latter is further expressed [28], where a significant difference was reported in the frequency of disagreement with other colleagues on the appropriate treatment plan between males and females. It is also reported that gender does not explain a statistically significant proportion of variability in the development of moral reasoning and that evidence does not support claims of gender polarity in moral orientations [38]. Thus, future research should concentrate on the psychological processes that give rise to moral maturity between genders [41]. Elsewhere, a small but significant gender-related difference in measures of ethical sensitivity, reasoning, and moral motivation was discussed [42]. Women were more sensitive and caring to patients’ needs, concerned, and empathetic.

Furthermore, women seemed to deal better with complex ethical problem issues and developed more effective action plans than men to resolve these situations. However, men and women did not differ significantly during the first years of their studies, but the change was apparent at the end of their studies. The most significant difference was in the proportion of women who demonstrated an ethical judgment profile suggesting greater responsibility in applying ethical deeds to resolve complex ethical issues [42]. Finally, the survey by McKenzie [43] showed that women demonstrated a greater responsibility in the dental profession than men in treating populations that are low-income, rural, and non-white. In the study of Behar-Horenstein et al. [44], male students scored significantly higher for personal interest and significantly lower for prioritizing ethical good in decision-making than female students.

Further, the study by Martinov-Bennie and Mladenovic [45] on undergraduate students demonstrated some variability connected to the type of ethical dilemma and some effects linked to gender. On the other hand, our findings showed that a statistically significant dilemma associated with gender appeared to be the dentist–patient relationship. Both male and female students seemed concerned about ethical challenges against their moral principles by 69.6% (2 out of 10 males and 5 out of 10 females). Elsewhere, no differences between male and female dentists were encountered amongst different ethical dilemmas [28,43].

Another factor affecting the ethical decisions of the dental students in Athens, Greece, was their place of origin. In contrast with certain stereotypes about urbanism [46], students of origin outside the capital were less interested in esthetic and quality dental services (for example, giving more than one treatment plan, being confronted with inadequate treatments from others, correcting another colleague’s mistake, interacting with other medical specialties, or being concerned with ethical dilemmas in the patient–dentist relationship). Elsewhere, in favor of the stereotype of differences between urban and non-urban environments, it is reported that people in more populous places emphasize on personal responsibility, individualism, determination, human acceptance, personal freedom, and communication [47], reflecting differences in estimation and dedication on high-quality dental services. As reported in Wirth’s seminal article on city life (1938) [48], urban society is more heterogeneous and exposure to different kinds of people promotes tolerance to communication differences that exceed satisfaction from quality services [46]. Materialism is reported to be primarily concentrated in cities where dental patients can be more consumerist and materialistic [49]; even vanity issues regarding one’s smile can contribute to different needs in the external picture of oneself, forcing dental students of this origin to be more interested in moral courage than their non-urban colleagues. Esthetic, luxury, positional, or conspicuous consumption is centered in the city, as discussed elsewhere [50]. 

For 5-year undergraduate dental students in this study, family income positively affected issues such as the cultivation of individual practice and clinical skills, trust issues, moral insight, and intuition, interaction with other medical professions, and ethical dilemmas in the patient–dentist relationship, suggesting that a wealthy background could positively affect future quality of dental services. Previous research that examines the relationship between personal finances and ethics argues that people from upper-class backgrounds behave more unethically in both the natural world and laboratory settings than lower-class individuals [51], with which we agree. The study of Wang and Murnighan [52] found that higher-income people are more likely to approve of unethical behavior than lower-income people in many countries.

Furthermore, following postgraduate studies, those 5-year undergraduate dental students in our study were more confronted with practices or policies inconsistent with the standard of care. At the same time, they also expressed more profound concern for patients who refuse to follow treatment. This could be attributed to greater dedication to excellence by people who continue education, as mentioned elsewhere [53], and the fact that they build their future work opportunities and income strategies to recompensate the investment of postgraduate studies on the extended and highly esthetic, thus more expensive, treatment plans. 

Conclusively, the null hypothesis of the present study was overall rejected as we found statistical differences in the influence that factors such as gender, place of origin, family income, and future educational and professional plans play in the formation of ethical manners of future dental professionals in Greece. As mentioned previously, the ability of a student to identify ethical issues and apply a reasonable decision-making plan to a fundamental ethical challenge is an ideal goal of ethics instruction [53,54,55,56,57,58]. The education of dental ethics should contribute to identifying a dilemma and taking the right actions to solve the issue under the principles of ethics that seem to present little difference at the international level since the same morals and values are also discussed elsewhere [1,5,6,7,59,60]. Thus, students and healthcare providers should own this knowledge to serve patients responsibly, excellently, and in the patient’s best interest. Considering the role of ethical values in constructing educational curricula worldwide, our data can lead to more ethical behavior practices among dental undergraduate students. Further, it will also contribute to overcoming the ethical challenges of the profession for young professionals working in different countries to those of their origin.

## 5. Conclusions

Five-year undergraduate dental students in Athens, Greece, show compassion for poor patients, respect patients’ autonomy, and guide patients to choose the best treatment plan before receiving dental ethics seminars. Female students are positively affected by integrity and altruism. However, they have difficulty conversing with colleagues. Origin outside the capital diminishes the significance of clinical skills, interaction with other medical specialties, and moral courage. Those expecting to follow postgraduate studies were more sensible against practices and policies of low standards and patients who refused treatment. At the same time, those who had other professional plans than dentistry was unwilling to reveal bad news to people. Presentations with clinical scenarios, multiple choice questions, and role-playing are the most preferred educational tools for teaching dental ethics.

## 6. Limitations of the Study

The study was applied only to one generation of dental students; thus, the results do not show the direction of causation, which may only be determined in the future—a more extended controlled experiment that would include participants from other generations. Additionally, the ethical behaviors analyzed here are based on data availability and omit other behaviors on financial transactions or communication issues. More data on the ethical behavior of Gen Z dentists should also be studied as Gen Z age. This would reveal whether the findings continue to strengthen as the cohort ages from 30 to 40 and then 50. Nevertheless, this early look at the specific data suggests that there appears to be a positive relationship between ethical footprints and gender, origin, family income, postgraduate studies, and future professional plans. These results have important implications for educators regarding dental ethics on an international level since ethics is an upcoming issue in all professional spheres, especially in health sciences [59,60]. Factors influencing undergraduate students’ decisions could be considered when planning relevant courses to incorporate ethics in the dental curriculum to a more extended level.

## Figures and Tables

**Table 1:** Descriptive data of the study.

Group	5th Year Students, N = 133
Gender	Male (33.8%) Female (66.2%)
Origin	Athens (48.1%) Urban (12.8%) Countryside (21.1%) Island (9.8%) Another country (8.3%)
Family Income	<15,000 EUR (14.3%) 15,000–25,000 EUR (20.3%) 25,000–35,000 EUR (15%) 35,000–50,000 EUR (15.8%) >50,000 EUR (12.8%) Not Knowing/do not care (21.8%)
Future MSc Studies	General dentistry (24.1%) Prosthetics (20.3%) Oral Surgery (19.0%) Orthodontics (10.5%) Stomatology (9.8%) Restorative (8.3%) Pedodontics (6.8%) Periodontology (6.0%) Endodontics (5.3%)
Plans	postgraduate studies (40.6%) dental employee (24.8%) work abroad (18.8%) private office (9.8%) another degree (4.5%) no dentistry (1.5%)

**Table 2:** Prevalence of common ethical dilemmas among dental students. (Never = never addressed the issue described, Rare = rarely addressed the issue, Often = often addressed the issue. Codes in front of the description of the dilemmas can be found in Appendix A in the description of the questionnaire).

Common Ethical Dilemmas	Never	Rare	Often
E1. Being confronted with inadequate treatment from other colleagues	9.8%	40.6%	49.6%
E2. Having to correct another colleague’s mistake	14.3%	50.4%	35.3%
E3. Disagreeing about treatment suggested by a colleague	16.5%	57.1%	26.3%
E4. Explaining your mistakes to the patient	31.6%	49.4%	18.8%
E5. Criticizing a colleague	72.2%	19.5%	8.3%
E6. Practices or policies that are not in line with the standard use of care	36.8%	44.4%	18.8%
E7. Over-treating a patient	60.9%	30.1%	9%
E8. Treating a patient with unreasonable demands	36.1%	36.1%	27.8%
E9. Dealing with a patient who asks you for inappropriate treatments	42.9%	33.8%	23.3%
E10. Working with a weak/incompetent decision maker	38.3%	48.1%	13.5%
E11. Working with a patient refusing treatment	38.3%	43.6%	18%
E12. Working with a patient with neglected oral hygiene by their guardian	23.3%	41.2%	35.3%
E13. Having to reveal bad news to your patient	36.8%	48.1%	15.1%
E14. Being confronted with a patient who criticizes a colleague for their prices	30.1%	38.3%	31.6%
E15. Dealing with a situation where the patient cannot pay off treatment	22.6%	48.1%	29.3%
E16. Failing to address ethical challenges	58.68%	31.6%	9.8%
E17. Violation of medical confidentiality	73.7%	23.3%	3%

**Table 3:** Presentation of the importance of values affecting dental students’ behavior. (No significance = represents those students that find the described moral as not significant, Little significance = those students that find this moral to be of little significance, Very significant = represents the students that find the described moral to be very significant. Codes in front of the description of the morals can be found in Appendix A in the description of the questionnaire).

Morals	No Significance	Little Significance	Very Significant
M1. Individual clinical practice and clinical skills	3.8%	15%	81.8%
M2. Social dentistry	4.5%	39.8%	55.6%
M3. Voluntary actions	9%	52.6%	38.3%
M4. Serving personal needs	13.5%	37.6%	48.9%
M5. Observance of the obligations and central values of the profession	2.3%	23.3%	74.4%
M6. Integrity	3%	22.6%	74.4%
M7. Trust	2.3%	14.3%	83.5%
M8. Altruism	6%	28.6%	65.4%
M9. Compassion	5.3%	28.6%	66.2%
M10. Justice	4.5%	21.1%	74.4%
M11. Moral courage	5.3%	22.6%	72.2%
M12. Moral insight	5.3%	24.1%	70.7%
M13. Interaction with other medical specialties	6%	23.3%	70.7%
M14. Difficult conversations with colleagues	5.3%	47.4%	47.3%
M15. Ethical dilemmas in the patient–dentist relationship	4.5%	26.3%	69.2%

**Table 4:** Statistically significant differences in responses vs. demographic factors. (Codes in front of the description of the morals can be found in Appendix A in the description of the questionnaire).

Factors	Statistically Significant Ethical Dilemmas and Parameters
Gender	M1. Individual practice and clinical skills (*p* = 0.032) M5. Observance of the obligations and central values of the profession (*p* = 0.002) M6. Integrity (*p* = 0.046) M7. Trust (*p* = 0.024) M8. Altruism (*p* = 0.032) M14. Difficult conversations with colleagues (*p* = 0.036) M15. Ethical dilemmas in the patient–dentist relationship (*p* = 0.024) *Female gender has a positive correlation with the above issues*.
Origin	C5. Mr. X, a 65-year-old active and good-looking man, came to your office to receive a new set of dentures. He believes that this will make him feel younger and he will be able to rebuild his life with his new partner. When the time comes to choose the color of his teeth, he says, “the whiter the better”. What would you do? (*p* = 0.007) C6. The dentist, studying all alternative treatment plans, gives the patient only one treatment option. What do you think? (*p* = 0.006) E1. Be confronted with inadequate treatments from other colleagues (*p* = 0.005) E2. Must correct another colleague’s mistake (*p* = 0.02) M1. Individual practice and clinical skills (*p* = 0.035) M4. Serving personal needs (*p* = 0.04) M11. Moral courage (*p* = 0.039) M13. Interaction with other medical specialties (*p* = 0.021) M15. Ethical dilemmas in the patient–dentist relationship (*p* = 0.07) *Students from outside the capital, Athens, consider the above issues less significant*.
Family Income	Q1. What is the most important ethical dilemma for you when practicing dentistry? (Which would trouble you as to whether to take over the incident or how to handle it?) (*p* = 0.029) M1. Individual practice and clinical skills (*p* = 0.003) M7. Trust (*p* = 0.008) M12. Moral insight and intuition (*p* = 0.02) M13. Interaction with other medical specialties (*p* = 0.03) M15. Ethical dilemmas in the patient–dentist relationship (*p* = 0.015) *Family income has a positive correlation with the above issues*.
Future MSc studies	C5. Mr. X, a 65-year-old active and good-looking man, came to your office to receive a new set of dentures. He believes that this will make him feel younger and he will be able to rebuild his life with his new partner. When the time comes to choose the color of his teeth, he says, “the whiter the better”. What would you do? (*p* = 0.017) E6. Being confronted with practices or policies that are inconsistent with the standard of care (*p* = 0.006) E11. Working with a patient refusing treatment (*p* = 0.047) E17. Violation of medical confidentiality (*p* = 0.023) M7. Trust (*p* = 0.039) M14. Difficult conversations with colleagues (*p* = 0.02) M15. Ethical dilemmas in the patient–dentist relationship (*p* = 0.016) *Future MSc studies exhibit a positive correlation with the above issues*.
Future plans	E13. Having to reveal bad news to your patient (*p* = 0.044) *Future professional plans have a positive correlation with the issue*.

## Abbreviations

Generation Z (Gen Z): People whose years of birth range from the mid-to-late 1990s to 2010–2012. Generation Χ (Gen Χ): People who were born between 1965 and 1981.

## Appendix A

Appendix A describes the questionnaire of the present study.

INTRODUCTORY MESSAGE

Dear colleagues,

This online survey aims to gather information on ethical dilemmas that you may have encountered during your clinical exercises at the dental school of the National and Kapodistrian University of Athens. It also aims to verify factors that influence responses to these ethical issues and give proposals of future effective educational approaches to the matter. Participation in the study is voluntary and no personal data are gathered. You have the right to refuse participation. No reward is given for your participation. You may confirm that you agree to complete the questionnaire by marking the box “Yes, I agree to participate”. Access to the questionnaire is only opened once. All questions must be answered to submit the form. Ethical approval has been obtained from the Ethics Board of the dental school, and the study proposal is registered under the number 395/20 April 2022.

PART ONE

1. What is your sex?

2. Which is your place of origin? Athens, Other Urban centre (prefecture capital), Continental periphery (towns and villages), Island region (towns and villages), Other country outside Greece.

3. What dental specialization would you like to pursue in the future? General dentistry, paedodontics, orthodontics, oral surgery dental surgery—aesthetic dentistry, endodontics, periodontology, stomatology/hospital dentistry, prosthodontics.

4. What is your family’s annual income?

R1. Less than 15,000 EUR.

R2. 15,001–25,000 EUR.

R3. 25,001–35,000 EUR.

R4. 35,001–50,000 EUR.

R5. Over 50,000 EUR.

R6. I do not know.

R7. We correctedIt is ok8. I have never been concerned about this issue.

5. What are you thinking of doing after graduation?

R1. I will open my own practice.

R2. I will work as an employee in a colleague’s office for some years until I decide what I will do.

R3. I will work as a dentist abroad.

R4. I will pursue postgraduate studies and at the same time, I will work in a colleague’s office.

R5. I will not practice dentistry.

R6. I will be occupied with something else professionally.

R7. I will continue my studies in another science.

PART TWO

Q1. What is the most important ethical dilemma for you when practicing dentistry? (Which would trouble you as to whether to take over the incident or how to handle it?)

R1. Your patient insists on extraction of a tooth against endodontic treatment for cost reasons.

R2. In an oral examination, you discover periodontal damage due to a poorly made restoration made by another colleague.

R3. You find suspicious damage to the oral mucosa and you need to inform your patient.

R4. A new patient seeking your services announces that he is HIV-positive.

R5. A young patient is experiencing long-term dental pain and the guardian did not seek your services earlier.

R6. You cannot manage a patient with an excessive fear of the dentist.

R7. Other.

Q2. When do you think the dentist can refuse their services?

R1. In a dental procedure that you feel you do not have the required training to perform.

R2. With patients who insist on irrational treatments/solutions.

R3. With financially unreliable patients.

R4. When your patient does not respect you and behaves inappropriately.

R5. With a patient with contagious infectious disease.

Q3. In your view, should the lifting of dental confidentiality be allowed?

R1. No.

R2. Yes, with prosecutorial intervention.

R3. Yes, among colleagues.

R4. Yes, when abuse is involved.

R5. Other.

Q4. Which of the following options would you consider as your role model in matters of moral character?

R1. Fellow students.

R2. University associates/professors.

R3. Qualified dentists.

R4. Media.

R5. Parents.

R6. Friends.

R7. Other.

Q5. Which method do you consider most effective in assessing students on ethical issues?

R1. Presentations with incidents involving ethical issues.

R2. Open-ended questions with scoring.

R3. Group role-playing game.

R4. Close-ended (multiple-choice) questions based on the code of ethics of the dental profession.

R5. Close-ended (multiple-choice) questions on the principles of biomedical ethics.

PART THREE

C1. A patient insists on mass extraction of his teeth against the conservative treatment that the dentist recommends to him. When the patient announces to his doctor that he will find another dentist for the extractions, then he decides not to treat him. What do you think?

R1. The dentist has the right to refuse to provide services.

R2. The dentist is indifferent to the patient’s choice.

R3. The behavior of the dentist is irrational.

R4. The dentist is not financially realistic.

C2. A 15-year-old patient came to your dental office. You have known her since she was a child as she and her parents have been coming to your office for years. When you had to take a panoramic X-ray, the patient saw the instruction that said that “in case of pregnancy the doctor should be informed”, she was alarmed, and finally revealed that she is pregnant but she is afraid and has not told her parents. What would you do?

R1. I would cancel the panoramic and recommend for her to talk to her parents.

R2. I would not take off the panoramic and ask for her parents’ consent by revealing the fact to them.

R3. I would not talk to her parents, I would urge her to talk to them herself and I would delay the treatment as much as I can.

R4. I would refer her to another dentist.

R5. I would postpone the appointment until I decide how I would handle the situation.

C3. Your patient wants all his remaining teeth extracted to put on complete dentures. You do not agree with this decision of the patient and do not want extractions because:

R1. It is assumed that the effects of this treatment will cause greater discomfort to the patient in the future.

R2. As a professional you only provide treatments in which you believe.

R3. You believe that raising patients’ awareness of dental issues will provide better results in the patient’s health.

C4. Your patient wants extraction of teeth that hurt him instead of the conventional treatment. You do not agree with the patient’s choice because:

R1. You are afraid that the patient will resort to another dentist less qualified than you to perform the extractions.

R2. You consider that the patient has the right to choose the treatment plan he wants.

R3. You believe that given the socio-economic profile of the patient this treatment is the best solution for him in the long run.

C5. Mr. X, a 65-year-old active and good-looking man, came to your office to make a new set of dentures. He believes that this will make him feel younger and he will be able to rebuild his life with his new partner. When the time comes to choose the color of his teeth he says, “the whiter the better”. What would you do?

R1. I show him the whitest shade in the color chart.

R2. I choose three shades that I think suit his features and age and tell him to choose one of them.

R3. I choose myself the shade that I consider best for his age and his face and tell him that if he does not agree he can go to another dentist.

R4. I give him the color chart and tell him to choose for himself what he likes.

R5. I try to convince him by performing the selection process in front of him without asking for his opinion.

R6. We work together on the choice and help each other until we come up with the final shade.

C6. The dentist, studying all alternative treatment plans, gives the patient only one treatment option. What do you think?

R1. All alternatives must be presented to the patient and they must be given the right to choose.

R2. The dentist must explain to the patient the different options in the treatment plans he has and guide them to the right choice.

R3. The dentist should only express an opinion if requested by the patient.

R4. The dentist is obliged to use their experience only for the benefit of the patient and therefore should only present to them what they consider appropriate.

C7. A patient asks to change the date and treatment provided in the dental insurance claim so that he can be compensated for his dental treatment. Explain to the patient that you cannot do this because:

R1. You have a moral obligation to be honest.

R2. You cannot discriminate against specific patients.

R3. You want to ensure sincere long-term cooperation with all your patients.

R4. There may be legal consequences.

C8. A 30-year-old male enters in an emergency your office with an oblique fracture in #11 reaching the pulp chamber. The patient has a free medical history and is unemployed. After a detailed clinical examination, you present to your patient the treatment plan that includes endodontic treatment of #11, restoration with a post and construction of a full-ceramic crown. He informs you that he would find it difficult to pay for the treatment of his tooth. What would you do?

R1. Pulpotomy to relieve the patient of pain and nothing more.

R2. You would refer your patient to a public service.

R3. You would only complete the endodontic treatment by facilitating the payment from the patient whenever that is possible.

R4. You would complete the entire treatment plan and facilitate the payment for the patient in due course.

C9. Mrs. X, 45 years old with three children and with a low income, has been your patient for 10 years. Three years ago, you built a metal–ceramic bridge of three pieces due to the lack of 36, which was paid off long after its construction. Last week, while Mrs. X was eating a sandwich, she found that the porcelain of the bridge was detached in two places. Although she was not in pain, she was aesthetically bothered by the inadequacy of porcelain and came to you angry. Although you explained to her that there are no guarantees for dental care, she still asks for pro bono replacement of the bridge. What would you do?

R1. Replacement of the bridge without payment.

R2. You would ask Mrs X to cover only the cost of the dental technician’s work.

R3. Mrs. X would have to pay 50% of the replacement cost.

R4. Mrs. X must pay for the construction of a new bridge as usual.

C10. The patient refuses to put the prosthetic work that you have made for him because he cannot speak and does not like them aesthetically. What would you do?

R1. You tell him that you have been charged for the work and therefore, he has to pay you even if he cannot bear it.

R2. You tell him that they are aesthetically acceptable even if you do not believe this is the case.

R3. You place them without asking for his opinion.

R4. You try to modify them to meet the objections of the patient.

R5. You remake them from scratch at your own expense.

R6. You remake them from scratch at the patient’s expense.

R7. You remake them from scratch at the expense of the dental technician.

R8. You refer him to another dentist.

R9. You work with an expert to finish the task.

E. In your academic career as a therapist, how often have you faced the following ethical dilemmas so far? 1 = never, 2 = rarely (1–2 times), 3 = often (4 and more times).

E1. Being confronted with inadequate treatments from other colleagues.

E2. Having to correct another colleague’s mistake.

E3. Disagreement about treatment suggested by a colleague.

E4. Explaining your mistakes to the patient.

E5. Criticizing a colleague.

E6. Being confronted with practices or policies that are inconsistent with the standard of care.

E7. Over-treating a patient.

E8. Treating a patient with unreasonable demands.

E9. Dealing with a patient who asks you for inappropriate treatments.

E10. Working with a weak or incompetent decision maker.

E11. Working with a patient refusing treatment.

E12. Working with a patient with neglected oral hygiene by their guardian.

E13. Having to reveal bad news to your patient.

E14. Being confronted with a patient who criticizes a colleague for their prices.

E15. Dealing with a situation where the patient cannot pay for treatment.

E16. Failing to address ethical challenges.

E17. Violation of medical confidentiality.

M. Which of the following issues do you consider important in terms of dental professionalism, in all the following scenarios and with what gradation? (Note a number from 1 to 3 (1 = not important at all, 2 = of little importance, 3 = very important).

M1. Individual practice and clinical skills.

M2. Social dentistry (in special groups of the population, poor, immigrants, people with disabilities, abused women, etc.).

M3. Voluntary social actions.

M4. Serving personal needs.

M5. Observance of the obligations and central values of the profession.

M6. Integrity.

M7. Trust.

M8. Altruism.

M9. Compassion.

M10. Justice.

M11. Moral courage.

M12. Moral insight and intuition.

M13. Interaction with other medical specialties.

M14. Difficult conversations with colleagues.

M15. Ethical dilemmas in the patient–dentist relationship.
